# GLUT10—Lacking in Arterial Tortuosity Syndrome—Is Localized to the Endoplasmic Reticulum of Human Fibroblasts

**DOI:** 10.3390/ijms18081820

**Published:** 2017-08-22

**Authors:** Alessandra Gamberucci, Paola Marcolongo, Csilla E. Németh, Nicoletta Zoppi, András Szarka, Nicola Chiarelli, Tamás Hegedűs, Marco Ritelli, Giulia Carini, Andy Willaert, Bert L. Callewaert, Paul J. Coucke, Angiolo Benedetti, Éva Margittai, Rosella Fulceri, Gábor Bánhegyi, Marina Colombi

**Affiliations:** 1 Department of Molecular and Developmental Medicine, University of Siena, 53100 Siena, Italy; alessandra.gamberucci@unisi.it (A.G.); paola.marcolongo@unisi.it (P.M.); benedetti@unisi.it (A.B.); rosella.fulceri@unisi.it (R.F.); 2 Department of Medical Chemistry, Molecular Biology and Pathobiochemistry, Semmelweis University, 1094 Budapest, Hungary; nemeth.csilla@med.semmelweis-univ.hu; 3 Division of Biology and Genetics, Department of Molecular and Translational Medicine, University of Brescia, 25123 Brescia, Italy; nicoletta.zoppi@unibs.it (N.Z.); nicola.chiarelli@unibs.it (N.C.); marco.ritelli@unibs.it (M.R.); g.carini001@unibs.it (G.C.); 4 Department of Applied Biotechnology and Food Science, Laboratory of Biochemistry and Molecular Biology, Budapest University of Technology and Economics, 1111 Budapest, Hungary; szarka@mail.bme.hu; 5 Department of Biophysics and Radiation Biology, Semmelweis University, 1094 Budapest, Hungary; tamas@hegelab.org; 6 Center for Medical Genetics, Ghent University, B-9000 Ghent, Belgium; Andy.Willaert@UGent.be (A.W.); Bert.Callewaert@UGent.be (B.L.C.); Paul.Coucke@UGent.be (P.J.C.); 7 Institute of Clinical Experimental Research, Semmelweis University, 1094 Budapest, Hungary; margittai.eva@med.semmelweis-univ.hu

**Keywords:** GLUT10, arterial tortuosity syndrome, dehydroascorbic acid, endoplasmic reticulum, nuclear envelope, Fe^2+^/2-oxoglutarate dependent dehydrogenases

## Abstract

GLUT10 belongs to a family of transporters that catalyze the uptake of sugars/polyols by facilitated diffusion. Loss-of-function mutations in the *SLC2A10* gene encoding GLUT10 are responsible for arterial tortuosity syndrome (ATS). Since subcellular distribution of the transporter is dubious, we aimed to clarify the localization of GLUT10. In silico GLUT10 localization prediction suggested its presence in the endoplasmic reticulum (ER). Immunoblotting showed the presence of GLUT10 protein in the microsomal, but not in mitochondrial fractions of human fibroblasts and liver tissue. An even cytosolic distribution with an intense perinuclear decoration of GLUT10 was demonstrated by immunofluorescence in human fibroblasts, whilst mitochondrial markers revealed a fully different decoration pattern. GLUT10 decoration was fully absent in fibroblasts from three ATS patients. Expression of exogenous, tagged GLUT10 in fibroblasts from an ATS patient revealed a strict co-localization with the ER marker protein disulfide isomerase (PDI). The results demonstrate that GLUT10 is present in the ER.

## 1. Introduction

*SLC2A* (solute carrier 2A) gene family encodes glucose transporter (GLUT) proteins; presently 14 proteins grouped into three classes upon their sequence similarities. GLUT transporters are overwhelmingly uniporters, with the exception of GLUT13, which has been described as a myo-inositol-H^+^ symporter. GLUT10, together with GLUT6, 8, 12 and 13, belongs to class 3, which is the least investigated group of GLUT transporters. Their substrates, subcellular distribution and physiological functions have been hardly characterized [1].

The mRNA of GLUT10 has been documented in human brain, heart, lung, liver, skeletal muscle, pancreas, placenta and kidney [2], as well as in mouse brain [3]. GLUT10 is abundantly expressed in human aortic vascular smooth muscle cells [4], which strengthen its role in the pathophysiology of ATS (arterial tortuosity syndrome). GLUT10 expressed in Xenopus oocytes exhibited 2-deoxy-d-glucose transport activity, which was inhibited by d-glucose and d-galactose [2]. Recently, GLUT10-mediated dehydroascorbic acid transport was also identified [5]. The intracellular presence of the protein was firstly reported by Coucke and co-workers [6].

GLUT10 protein is encoded by the *SLC2A10* gene [1]. Loss-of-function mutations of the *SLC2A10* gene are responsible for arterial tortuosity syndrome (ATS, Online Mendelian Inheritance in Man compendium-OMIM-#208050), which is a monogenic autosomal recessive heritable connective tissue disorder. The clinical phenotype of ATS includes the elongation, tortuosity and aneurysms of large arteries and stenosis of the pulmonary artery. To date, various *SLC2A10* gene mutations resulting in ATS have being identified [6,7,8,9,10,11]. However, the precise subcellular localization of GLUT10, and the missing physiological role of the transporter that results in the appearance of the ATS phenotype still remain debated. Three hypotheses have been proposed to connect the absence of GLUT10 activity and the connective tissue defects of ATS, as described in details in a previous paper from our laboratories [5]. Briefly, it was originally proposed that GLUT10 is a glucose transporter in the nuclear envelope (NE). Glucose-dependent upregulation of decorin, a transforming growth factor beta (TGFβ) signaling inhibitor, would be absent in ATS, which also explains the activation of TGFβ pathway observed in ATS fibroblasts [6]. The second hypothesis supposes that GLUT10 mediates the transport of dehydroascorbic acid (DAA, the oxidized form of the antioxidant ascorbic acid, AA) across the mitochondrial inner membrane [12]. DAA can be reduced to AA in the mitochondrial matrix, which can neutralize reactive oxygen species (ROS) and protects the cell from oxidative effects. A third hypothesis postulates that GLUT10 behaves like a DAA transporter in the endoplasmic reticulum (ER) membranes [13,14]. DAA, upon being transported into the ER lumen, is reduced to AA and serves as a cofactor for Fe^2+^/2-oxoglutarate dependent dioxygenases. These enzymes catalyze the hydroxylation of prolyl and lysyl residues by reactions crucial for maturation and folding of several extracellular matrix proteins. Moreover, GLUT10 in the ER-derived NE might favor the entry of DAA in the nucleus working as a cofactor (after reduction to AA) for nuclear Fe^2+^/2-oxoglutarate-dependent dioxygenases involved in epigenetic modifications of histones and nucleic acids as hypothesized in [15].

In a recent paper, we observed that DAA uptake is present in the intracellular compartments of human normal fibroblasts, but dramatically diminished in fibroblasts from ATS patients [5]. Moreover, we demonstrated that in vitro-translated GLUT10 protein mediates DAA transport in proteoliposomes [5]. These observations suggest that GLUT10 can transport DAA and indicate its localization in cellular endomembranes.

Since there is no consensus with respect to the subcellular distribution of GLUT10, the aim of the present study was to unambiguously clarify the localization of this transporter. Obviously, the intracellular position of GLUT10 is crucial for further definition of its function and the clarification of ATS pathogenesis.

## 2. Results

### 2.1. In Silico Prediction of Subcellular Localization of GLUT10

An anticipatory in silico search for the prediction of the subcellular distribution of GLUT10 indicated a relatively high probability of ER localization and an absolutely low probability of mitochondrial occurrence for GLUT10 (Table 1). Moreover the coding sequence for human GLUT10 possesses the ER retention signal YXXI/V motifs in C-terminus [16] and a Lys-Arg-Arg (KRR) motif in C-terminus that is also an ER retention signal [17].

### 2.2. GLUT10 Is Predominantly Present in the Microsomal Fraction

**Figure 1:**
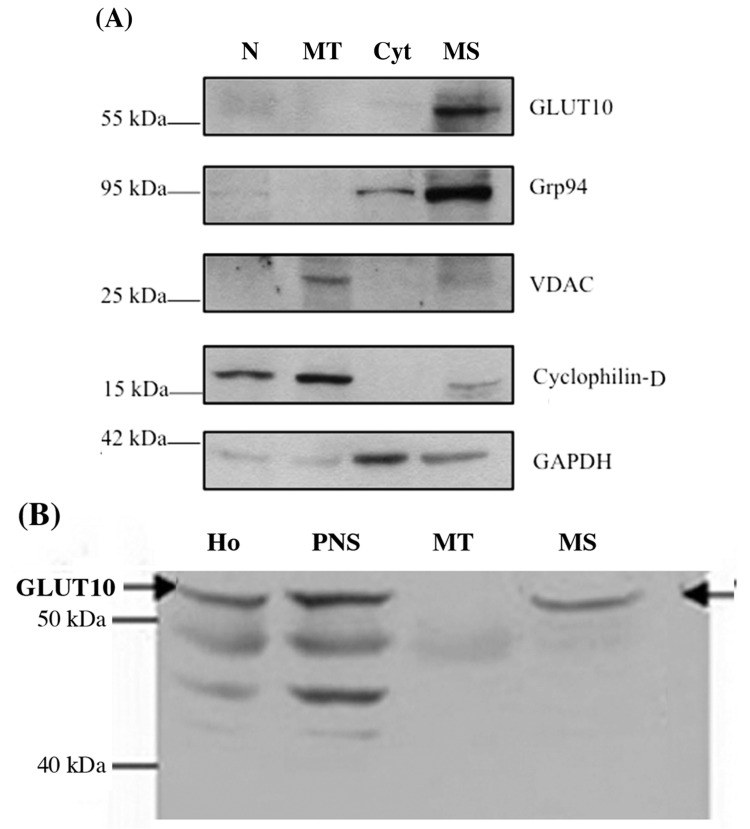
GLUT10 is present in the microsomal fraction of human fibroblasts and liver tissue. Subcellular fractions were prepared from human skin fibroblasts (**A**) or human liver tissue (**B**) and analyzed by Western blot after SDS-page separation of proteins, with antibodies to the GLUT10 protein as well as the marker proteins Grp94 for microsomes, VDAC1 and cyclophilin D for mitochondria, and GAPDH for the cytosol, as detailed in the “Experimental” section. Abbreviations: N: Nuclei; MT: mitochondria; Cyt: cytosol; MS: microsomes; Ho: homogenate; PNS: post nuclear supernatant.

In a first set of experiments the subcellular localization of GLUT10 was investigated on fractions prepared by differential centrifugation. Immunoblotting of the subcellular fractions derived from human control fibroblasts revealed that the GLUT10 protein was present in the microsomal fraction (ER-derived), whilst virtually absent in the mitochondrial fraction (Figure 1A). The fraction identity was validated by the presence of suitable marker proteins, in particular the ER marker Grp94 was largely associated to the same microsomal fraction, while the mitochondrial and cytoplasmic markers—VDAC1/cyclophilin D and GAPDH, respectively—were absent or only slightly present in the microsomal fraction (Figure 1A). To further support these findings, the subcellular distribution of GLUT10 was also immunodetected in conventionally prepared liver subcellular fractions. Again, the immunoreactive GLUT10 protein was present in the microsomal but not in the mitochondrial fraction prepared from human liver (Figure 1B).

### 2.3. GLUT10 Immunofluorescence Reveals a Reticular Pattern in Control but Not in ATS Fibroblasts

**Figure 2:**
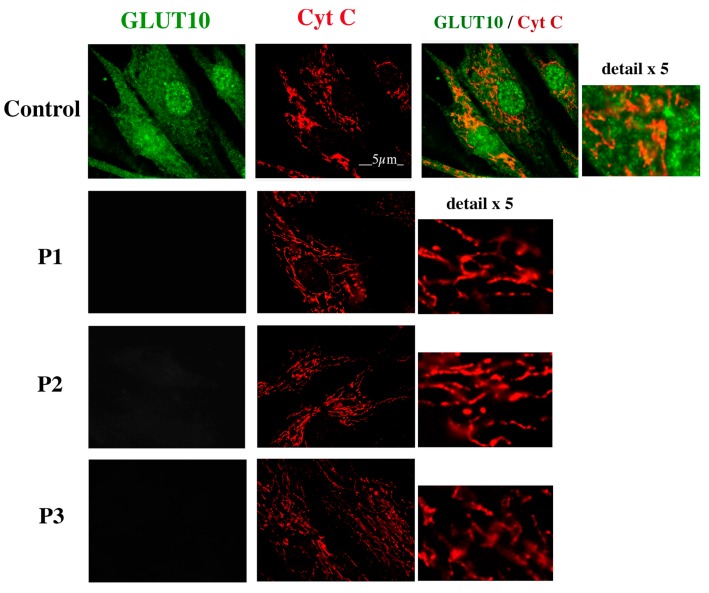
GLUT10 and mitochondrial immunostaining in fibroblasts from human healthy subjects and three arterial tortuosity syndrome (ATS) patients. Fibroblasts were prepared and immunoreacted with the anti-GLUT10 antibody Ab1 and an antibody to Cyt C, and the images were acquired by fluorescent microscopy as detailed in the “Experimental” section. P1, P2 and P3 indicate three unrelated ATS patients as described in the “Experimental” section. Scale bar = 5 μm.

Immunofluorescence of human control fibroblasts showed a reticular distribution of the protein and a perinuclear abundance of GLUT10 (Figure 2), as observed in previous studies [6,18]. The possible presence of GLUT10 in the mitochondrial network was investigated by localizing mitochondria with the immunoreaction of the cytochrome c protein (Cyt C). The decoration patterns of GLUT10 and of mitochondria were very different (see also enlarged detail in Figure 2). As also shown in Figure 2, GLUT10 could not be detected at all in fibroblasts from the three unrelated ATS patients [6,9,18], while Cyt C decoration was present in a pattern similar to control cells.

**Figure 3:**
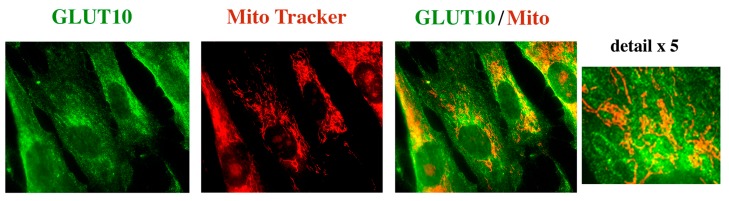
GLUT10 immunostaining and mitochondrial decoration with a fluorescent probe in the human fibroblast cell line BJ-5ta. Human fibroblast immortalized with hTERT (BJ-5ta line) were immunoreacted with the anti-GLUT10 antibody Ab2, and the mitochondrial fluorescent probe Mito Tracker™ Orange as reported in the “Experimental” section. Images were acquired by fluorescent microscopy as detailed in the “Experimental” section. Scale bar = 5 μm.

To further prove the lack of GLUT10 localization to mitochondria, human skin fibroblasts immortalized with human telomerase reverse transcriptase (hTERT) were treated in vivo with a mitochondrial fluorescent probe, and subsequently fixed and immunoreacted for GLUT10. As shown in Figure 3, GLUT10 and mitochondria presented with a very different decoration pattern.

### 2.4. GLUT10 Co-Localizes with the ER Marker Protein PDI

**Figure 4:**
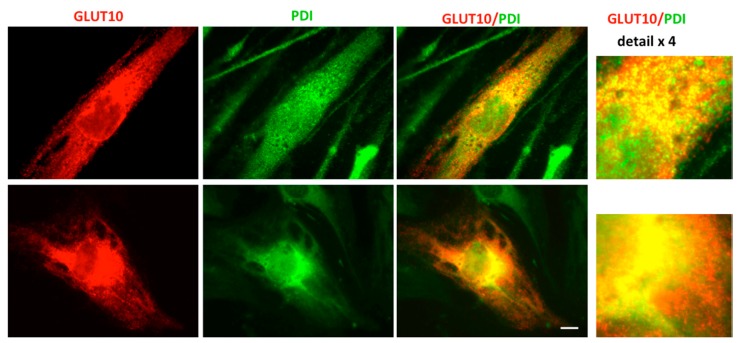
GLUT10 co-localizes with the ER marker protein disulfide isomerase (PDI). Tagged GLUT10 was transiently transfected in fibroblasts of the ATS patient P1 that were immunoreacted with antibodies to the GLUT10 tag and the ER marker PDI as reported in the “Experimental” section. The images were acquired by fluorescent microscopy as detailed in the “Experimental” section. Scale bar = 10 μm.

Our previous efforts for the demonstration of GLUT10 co-localization with an ER marker have failed. Although antibodies to several ER markers have been tested, GLUT10 immunoreaction somehow quenched the labeling of the ER marker proteins. To overcome this problem, tagged GLUT10 was transiently transfected into fibroblasts of the ATS patient P1. This approach resulted in the decoration of both tagged GLUT10 and the ER marker protein disulfide isomerase (PDI) (Figure 4). An evident co-localization of GLUT10 and PDI was present with an intense perinuclear decoration (see also details in Figure 4).

## 3. Discussion

Pathogenetic mechanisms underlying connective tissue disorganization have not been clarified in details in ATS, in spite of the unequivocally identified mutations in *SLC2A10* gene encoding GLUT10 transporter as the genetic cause of the disease. The elucidation of subcellular localization of GLUT10 is crucial for further clarifying the pathomechanism of ATS. The results reported here demonstrate that GLUT10 localizes to the ER and much likely in other elements of the endomembrane system of human fibroblasts. Several lines of evidence support our conclusion.

First, in silico data for the GLUT10 coding sequence suggest ER localization and appear to exclude a mitochondrial one (Table 1).

Second, immunoblotting of the GLUT10 protein revealed its presence in the microsomal (ER-derived) fractions obtained from human fibroblasts and liver tissue, but not in the mitochondrial fractions (Figure 1). The role of GLUT10 in liver pathophysiology—acting either as a DAA or a glucose transporter—is the object of an ongoing study in our laboratories; in any event, the lack of pathology in ATS liver might be due to the presence of multiple DAA/glucose transporters in the liver ER membrane [19].

Third, the immunoreaction of GLUT10 in human fibroblasts revealed an even distribution along the cytosol, with a more intense perinuclear representation of the reactive protein (Figure 2), which strongly suggests ER localization. On the other hand, the mitochondrial network, revealed with both anti-Cyt C antibodies or a mitochondrial probe, presented with a completely different morphology (Figure 2 and Figure 3). The nuclear decoration in control fibroblasts, but not in the hTERT-fibroblast line, might be unspecific as GLUT10 protein was also absent by Western blotting in the fibroblast nuclear fraction. Nonetheless, we cannot fully exclude the decoration of the nucleoplasmic reticulum, which has been reported in many cell types including fibroblasts [20]. In addition to the cell difference, the immunostaining protocol and the antibody to GLUT10 were different in the case of short-term cultured fibroblasts (Figure 2) and hTERT-immortalized fibroblast line (Figure 3). In any event, the immunoreaction protocol applied to human fibroblasts (widely used in previous studies) should unequivocally decorate GLUT10, since it did not reveal any signal in three ATS patients (Figure 2), but again revealed the protein after *SLC2A10* re-expression in fibroblasts of ATS patients [5]. The lack of immunodetectable GLUT10 protein in the ATS fibroblasts can be easily explained in P1, as his mutations results in the lack of protein expression. The *SLC2A10* gene mutations of P2 can theoretically result in a lower expression of the GLUT10 protein with a single amino acid substitution. P3 patient should theoretically produce a smaller protein, as the exon 3-derived amino acid sequence is absent. The logical explanation for these observations is that the modified proteins or their mRNAs are eliminated by a sort of cell quality control [21].

Fourth, the immunorevelation of tagged GLUT10 expressed in ATS fibroblasts from P1 and of the ER classic marker PDI showed a strict co-localization of GLUT10 and PDI (Figure 4). Again, a perinuclear localization of GLUT10 was evident, and the localization pattern was similar to that reported in rat aortic smooth muscle cells transiently expressing the green fluorescent protein-labeled transporter by another author [13].

The original paper of the field already suggested a perinuclear abundance of GLUT10 in human fibroblasts, deciphered as localization in the NE [6]. However, a subsequent study reported that GLUT10 was present in mitochondria and the Golgi apparatus in smooth muscle cells and insulin-stimulated adipocytes of mice [12].

Our results militate against the mitochondrial localization of GLUT10 [12]. This discrepancy might be due to species differences (human in the present study versus mouse in reference 12), and/or to the presence of mitochondria-associated ER membranes (MAMs) [22] in the mitochondrial fractions. It was observed that GLUT10 knock-out mice do not present an evident ATS pathology [8], which makes the mouse model pretty different. Moreover, mitochondria are equipped with several other AA and DAA transporters [23,24,25,26], which questions the importance of GLUT10 as a mitochondrial transporter [27]; consistently, we observed a similar mitochondrial DAA uptake in control and ATS fibroblasts As to the reported localization of GLUT10 to the Golgi [12], it was observed in murine 3T3-L1 cell line only, but not in rat A10 smooth muscle cells, and therefore species/cell-type differences might account for these discrepancies. Although we cannot fully exclude the presence of the transporter also in the Golgi of human fibroblasts, the even distribution of GLUT10 indicates a widespread ER localization being the Golgi only a minor portion of the endomembrane network.

The function of DAA and/or AA that is missing in the ER lumen of fibroblasts from ATS patients is still enigmatic. Besides the plausible antioxidant role, AA serves as a cofactor of Fe^2+^/2-oxoglutarate-dependent dioxygenases [28]. This group of the enzymes includes hydroxylases involved in the posttranslational modification of collagen, elastin and other extracellular matrix proteins in the ER [29]. Thus, shortage of AA in the ER luminal compartment can depress the formation of extracellular matrix proteins. Moreover, GLUT10 in the ER-derived NE by allowing the entry of DAA in the nucleoplasma can regulate the activity of various nuclear Fe^2+^/2-oxoglutarate-dependent dioxygenases, which catalyze the demethylation of histones and nucleic acids as well as the hydroxylation of certain histones and also participate in DNA repairing processes [15]. Together, such epigenetic and posttranslational events can result in the pathological changes of ATS fibroblasts [15]. In accordance with this assumption, altered gene expression has been observed in a recently published transcriptome analyses performed in ATS fibroblasts [18]. The reported changes affected the expression of numerous genes involved not only in TGFβ signaling and extracellular matrix formation, but also in specific pathways controlling bioenergetics and oxidative stress response [18]. In summary, the present results and our previous findings [5,18] reinforce the hypothesis that ATS is a vitamin C compartmentalization disease [13,15]. Further studies are needed to clarify the molecular details of subcellular mechanisms impaired by AA/DAA depletion in these intracellular compartments.

## 4. Materials and Methods

### 4.1. In Silico Analysis

The sequence of GLUT10 was retrieved from the Uniprot database (http://www.uniprot.org/). Prediction softwares used: Target P: http://www.cbs.dtu.dk/services/TargetP/ [30]; Mitoprot II: http://ihg.gsf.de/ihg/mitoprot.html [31]; Predotar: https://urgi.versailles.inra.fr/predotar/predotar.html [32]; Psort II: http://psort.hgc.jp/form2.html [33]; MultiLoc/TargetLoc: http://abi.inf.uni-tuebingen.de/Services/MultiLoc/ [34]; ngLOC: http://genome.unmc.edu/ngLOC/index.html [35]; YLoc: http://abi.inf.uni-tuebingen.de/Services/YLoc/webloc.cgi [36] and CELLO v2.5: http://cello.life.nctu.edu.tw/ [37]. The reliability of the prediction tools has been double-checked by testing GLUT1-4 proteins and the ER marker protein CYP2E1

### 4.2. Fibroblast Culture

Skin fibroblasts from three ATS patients and unrelated healthy donors were established from skin biopsies as previously reported [6,18]. The ATS patients were: patient 1 (P1) homozygous for the c.1334delG microdeletion [6]; patient 2 (P2) compound heterozygous for the c.1309G > A and the c.1330C > T transitions [7]; patient 3 (P3) homozygous for the c.1411+1G > A splicing mutation [9]. Written informed consent was obtained from the patients and healthy individuals for skin biopsy procedure. This study was approved by the medical ethical committee of the University Hospital Spedali Civili of Brescia, Italy, and was performed in accordance with the Declaration of Helsinki Principles. A human skin fibroblast cell line immortalized with hTERT (BJ-5ta) was purchased from LGC Standards (Teddington, Middlesex, UK).

Fibroblasts from healthy subjects and ATS patients were grown in vitro at 37 °C in a 5% CO_2_ atmosphere in Earle’s Modified Eagle Medium (MEM) supplemented with 2 mM l-glutamine, 10% fetal bovine serum, 100 μg/mL penicillin and streptomycin (Life Technologies, Carlsbad, CA, USA). Cells were analyzed at the same in vitro passage number (5th to 7th). BJ-5ta fibroblasts were grown in a 4:1 mixture of MEM and Medium199 supplemented with 0.01 mg/mL hygromycin B (Life Technologies, Carlsbad, CA, USA), 10% fetal bovine serum and 1% Antibiotic-Antimycotic (Life Technologies). All cultures were grown in humidified incubator at 37 °C with a 5% CO_2_ in air atmosphere.

### 4.3. Subcellular Fractionation of Human Fibroblasts

Subcellular fractions were prepared from human control fibroblasts as reported earlier [38] with minor modifications. Cell homogenates were centrifuged for 10 min at 1000× *g*. The postnuclear supernatants were centrifuged for 20 min at 18,000× *g* to obtain mitochondrial fraction. Microsomes were recovered by ultracentrifugation for 60 min at 195,000× *g*. Pellets were resuspended in 20 mM 3-(*N*-morpholino)propanesulfonic acid (MOPS) buffer (pH 7.2) and maintained in liquid N_2_.

### 4.4. Preparation of Subcellular Fractions from Human Liver

Subcellular fractions from human liver were performed as described earlier [39]. The human liver samples were portions of tissue obtained under surgical operation at the Semmelweis University. The liver samples were graded by a pathologist on routine histochemistry on a scale of 1−5, and only liver samples graded 1 (1 being apparently normal and 5 severely diseased) were used.

### 4.5. Western Blot Analysis

Western blots were carried out as described previously [39]; 20 μg protein was loaded from each fraction. The membranes were incubated with antibodies against GLUT10 (1:1000, Abcam, Cambridge, UK), voltage dependent anion channel 1 (VDAC1) (1:1000; Santa Cruz Biotechnology, Inc., Dallas, TX, USA), cyclophilin D (1:2000; MitoSciences, Eugene, OR, USA), Grp94 and glyceraldehyde-3-phosphate dehydrogenase (GAPDH) (1:5000; Santa Cruz Biotechnology, Inc., Dallas, TX, USA).

### 4.6. Construction of a Tagged-pG10 Expression Vector

Ten nanograms of the pG10 expression vector [5,18] were used as PCR template to amplify the full-length coding sequence of *SLC2A10*, from the Kozak consensus sequence to the last amino acid codon. The PCR product was gel-purified and directly inserted into the pEF6/V5-His-TOPO™ expression vector according to the manufacturer’s instructions (Life Technologies, Carlsbad, CA, USA). Prior to transfection, the pG10-tag plasmid was sequenced to verify the correct in-frame insertion of GLUT10 with the C-terminal V5-His amino acids present on the expression vector. Transient transfection of pG10-tag into skin fibroblasts of the ATS patient P1 was achieved using the TurboFect transfection reagent in accordance with the manufacturer’s instructions (Thermo Scientific, Waltham, MA, USA).

### 4.7. Immunofluorescence Microscopical Analysis

To immunoreveal the GLUT10 protein, control and ATS fibroblasts were grown 48 h and reacted for 2 min with 3% paraformaldehyde/0.5% Triton, 20 min with 3% paraformaldehyde, washed with 100 mM glycine/phosphate buffered saline (PBS), blocked for 30 min with 5% bovine serum albumin (BSA) and immunoreacted overnight at +4 °C with 20 μg/mL rabbit polyclonal anti-human GLUT10 antibody (Alpha Diagnostic Int. Inc., San Antonio, TX, USA), referred to here as Ab1. After washing in PBS, cells were incubated for 1 h at room temperature with 1:1000 anti-rabbit secondary antibody conjugated to Alexa Fluor 488. The double staining was performed immunoreacting with 2 µg/mL anti-Cyt C monoclonal antibody (clone 6H2-B4) for 2 h and with an anti-mouse secondary antibody conjugated to Alexa Fluor 594. In certain experiments, human BJ-5ta fibroblasts grown on cover slides were incubated with 200 nM Mito Traker™ Orange (Molecular probes, Thermo Fisher Scientific, Waltham, MA, USA) for 15 min at 37 °C, washed and fixed with 4% paraformaldehyde for 15 min, treated with cold (−20 °C) acetone, washed with PBS, blocked for 30 min with 2% BSA and immunoreacted overnight at +4 °C with rabbit polyclonal anti-human GLUT10 antibody (1:200, Abnova, Walnut, CA, USA), referred to here as Ab2. After washing in PBS, cells were incubated for 1 h at room temperature with 1:2000 anti-rabbit secondary antibody conjugated to Alexa Fluor 488.

The signals were acquired by a cooled digital camera, DS Qi1, (Nikon, Japan) mounted on a Nikon Eclipse Ti inverted fluorescence microscope. The experiments were repeated three times.

The co-localization of GLUT10 with the protein disulfide isomerase (PDI) was analyzed in the ATS fibroblasts from P1 by transient transfection of tagged GLUT10. Fibroblasts grown for 48 h were fixed in cold methanol and immunoreacted for 2 h with 1:100 rabbit polyclonal anti-PDI antibody (Novus Biologicals, Littleton, CO, USA), which labels ER, and 1 µg/mL anti-V5 monoclonal antibody (Sigma Chemicals, St. Louis, MO, USA) to label tagged GLUT10. Cells were incubated for 1 h with 1:1000 anti-rabbit and anti-mouse secondary Abs conjugated to Alexa Fluor^®^ 488 and 594, respectively. Immunofluorescence signals were acquired by a CCD camera (SensiCam-PCO Computer Optics GmbH, Kelheim, Germany) mounted on Zeiss fluorescence Axiovert microscope (Zeiss, Oberkochen, Germany). The experiments were repeated three times.

## Figures and Tables

**Table 1:** Subcellular localization of GLUT10 predicted by in silico analysis.

Location	Target P	Mitoprot	Predotar	PSORT II	MultiLoc/TargetLoc	ngLOC	yLoc	Cello
	Probability of Location							
Plasma membrane	-	-	-	43.5%	0.12	14.46	99.8%	4.855
Endoplasmic reticulum	0.982	-	0.89	39.1%	0.63	-	0.1%	0.008
Extracellular space	-	-	-	4.3%	0.03	-	0.1%	0.061
Lysosome	-	-	-	-	0.06	-	0.0%	0.029
Golgi apparatus	-	-	-	4.3%	0.14	-	0.0%	-
Peroxisome	-	-	-	-	0.01	-	0.0%	0.007
Mitochondrion	0.014	0.0097	0.00	4.3%	0.0	-	0.0%	0.009
Cytoplasm	-	-	-	-	0.0	40.73	0.0%	0.010
Nucleus	-	-	-	-	0.0	8.909	0.0%	0.004

The output of each prediction software is an estimated probability or prediction score that correlates with the probability of the predicted localization. The higher the output scores the higher the probability that the protein (GLUT10) is localized in the certain compartment. Empty cells: the prediction tool does not give probability score for this localization.

## Abbreviations

AA	Ascorbic acid
ATS	Arterial tortuosity syndrome
BSA	Bovine serum albumin
Cyt C	Cytochrome C
DAA	Dehydroascorbic acid
ER	Endoplasmic reticulum
GAPDH	Glyceraldehyde-3-phosphate dehydrogenase
GLUT	Glucose transporter
hTERT	Human telomerase reverse transcriptase
KRR	Lys-Arg-Arg
MOPS	3-(*N*-morpholino)propanesulfonic acid
NE	Nuclear envelope
PBS	Phosphate buffered saline
PDI	Protein disulfide isomerase
TGFβ	Transforming growth factor beta
VDAC	Voltage dependent anion channel
